# Understanding the Existence of a Na_2_ Dimer in a High-Spin State

**DOI:** 10.1021/acs.jpca.5c03939

**Published:** 2025-09-02

**Authors:** Mehmet Emin Kilic, Puru Jena

**Affiliations:** Physics Department, 6889 Virginia Commonwealth University, Richmond, Virginia 23284, United States

## Abstract

The recent observation of a high-spin Na_2_ dimer formed on the surface of liquid helium nanodroplets raises some fundamental questions, as the ground state of Na_2_ is known to have zero spin. Is it protected against spontaneous dissociation? What is its binding energy and interatomic distance? Is it stable at a higher temperature? Using calculations based on density functional theory (with and without long-range interaction) and coupled cluster methods, CCSD­(T), we show that the bonding in the high-spin Na_2_ dimer is governed by van der Waals interaction with binding energy (bond length) varying between −0.030 eV (5.108 Å) and −0.192 eV (4.231 Å), depending on the computational method used. Thus, the experimental method used by Kresin and co-workers can be very useful to study larger metastable high-spin clusters such as Li_4_ which was predicted in 1985 to have a tetrahedral structure carrying a magnetic moment of 2 μ_B_, while its ground state is planar and nonmagnetic.

## Graphical abstract

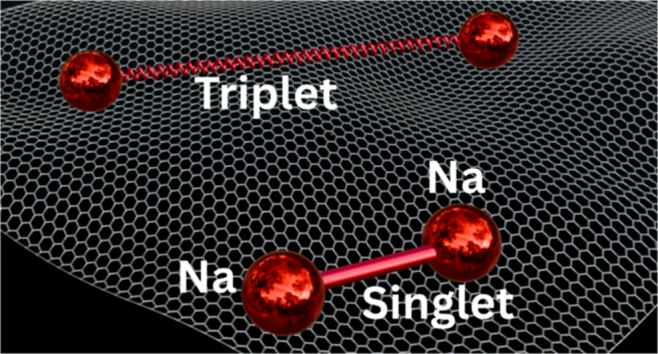

## 1 Introduction

Unlike transition metal clusters,^1^ alkali metal clusters tend to have low spins^2^ with even-numbered clusters having zero spin (singlet) and odd-numbered ones carrying a magnetic moment of 1 μ_B_ (doublet). Some 40 years ago, one of the authors of this paper predicted that the Li_4_ cluster, which is planar and has zero spin in its ground state, could have a magnetic moment of 2 μ_B_ (triplet) if it assumes a tetrahedral configuration.^2,3^ The triplet tetrahedral Li_4_, although metastable, lies lower in energy than a singlet Li_4_ having the same tetrahedral geometry. This interplay between the geometry and magnetism of the Li_4_ cluster is yet to be verified experimentally.

A decade later, the observation of spin triplet Na_2_ dimer and spin quartet Na_3_ trimer on the surface of superfluid helium nanodroplets renewed interest in the exploration of metastable alkali clusters with high spins.^4–6^ In a recent experiment,^7^ by measuring the magnetic deflection of Na_2_ dimer on helium nanodroplets, Kresin and co-workers provided direct and unambiguous evidence that it carries a magnetic moment of 1.9 ± 0.3 μ_B_. The authors argued that the weakly bound triplet Na_2_ dimer on helium nanodroplets remains in metastable form, while “the highly exothermic formation of the singlet ground state of Na_2_ is apt to eject it from the droplet”. This raises some fundamental questions. Does the metastable state correspond to a minimum in the potential energy landscape, thus preventing it from spontaneous dissociation? What is its binding energy, and how far apart are the Na atoms compared to its ground state structure? Does the triplet Na_2_ lie lower in energy than the singlet Na_2_ at the same interatomic distance? How stable is the metastable dimer at a higher temperature? In this work, we address these questions.

## 2 Computational Methods

We performed calculations using density functional theory (DFT) with B3LYP and PBE0 functionals with and without including Grimme’s dispersion (D3) correction, as well as the coupled cluster method with singles, doubles, and perturbative triples [CCSD­(T)].^8^ Various basis sets were employed, including def2-TZVP, cc-pVTZ, and aug-cc-pVTZ, as implemented in the Gaussian16 software package.^9^ We note that the CCSD­(T) is widely regarded as the “gold standard” for accurately capturing the electronic correlation effect, including long-range interactions. All calculations utilized very tight self-consistent field (SCF) convergence criteria and strict geometry optimization thresholds to ensure numerical stability and accuracy. For open-shell systems such as the triplet state of Na_2_, we used an unrestricted Hartree–Fock (UHF) reference. We verified the spin expectation value of <*S* ^2^> = 2.00, confirming negligible spin contamination in these cases. The basis set superposition error (BSSE) was assessed using the counterpoise method^10^ at both the DFT and CCSD­(T) levels. The BSSE was found to be small with the cc-pVTZ basis set [0.002 eV at the DFT level and 0.008 eV at the CCSD­(T) level]. To assess basis set convergence, calculations were performed using the Dunning correlation-consistent basis sets (cc-pVXZ, where X = D, T, Q) as well as their augmented counterparts (aug-cc-pVXZ). As shown in Table S1 of the Supporting Information, the energy differences beyond X = T are very small, indicating satisfactory convergence (see also Figure S1 in the Supporting Information). To evaluate the effect of additional diffuse functions, we compared total energies calculated by using the daug-cc-pVXZ and taug-cc-pVXZ basis sets with those obtained from the standard aug-cc-pVXZ sets. As summarized in Table S1, the energy differences between aug-cc-pVQZ and daug-cc-pVQZ were found to be negligible, suggesting that the inclusion of extra diffuse functions has an insignificant impact on the total energy.

## 3 Results and Discussion

The interatomic distances, binding energies, and HOMO–LUMO energies obtained from these methods for the ground state Na_2_ in a spin-singlet configuration are summarized in Table 1. The binding energy (*E*_B_) was calculated using the expression *E*_B_ = *E*(Na_2_) – 2*E*(Na), where *E*(Na) is the total energy of an isolated Na atom and *E*(Na_2_) is the total energy of the Na_2_ dimer. Note that in this definition, a negative binding energy implies that Na_2_ is bound. In agreement with previous calculations,^11,12^ the ground state of Na_2_ dimer is found to be a spin singlet with an equilibrium interatomic distance of 3.043–3.102 Å and a binding energy of −0.695 to −0.805 eV, depending on the theoretical methods used. These results are in good agreement with experimental values, which report a dissociation energy of −0.74 eV and a bond length of 3.08 Å.^12^ Moreover, the electronic structure analysis of the Na_2_ dimer reveals that the HOMO–LUMO gap ranges from 2.04 to 4.58 eV, corresponding to the energy difference between the bonding σ­(3s) and antibonding σ*­(3s) molecular orbitals (Figure 1). These values are notably smaller than the 5.42 eV separation between the 3s and 3p atomic orbitals in the isolated sodium atom. This reduction in the HOMO–LUMO gap upon dimerization suggests increased electronic delocalization and stabilization of the Na_2_ system compared with the isolated atom. The narrowing of the energy gap can be associated with bonding interactions and partial orbital overlap between the two sodium atoms, which facilitates electron delocalization and slightly raises the energy of the LUMO while lowering that of the HOMO relative to the atomic case.

**Table 1:** Calculated Bond Distances (Å), Binding Energies (eV), HOMO–LUMO Energies (eV), and HOMO–LUMO Gaps (eV) for the Na_2_ Dimer (Singlet) Using DFT (B3LYP-D3 and PBE0-D3) and CCSD­(T) Methods^a^

	DFT(B3LYP)-D3			DFT(PBE0)-D3			CCSD(T)
	def2tzvp	cc-pvtz	aug-cc-pvtz	def2tzvp	cc-pvtz	aug-cc-pvtz	cc-pvtz
*R*_Na–Na_	3.054	3.043	3.040	3.102	3.091	3.090	3.078
*E*_B_	–0.781	–0.770	–0.772	–0.710	–0.695	–0.696	–0.805
*E*_HOMO_	–3.559	–3.550	–3.554	–3.604	–3.603	–3.605	–4.522
*E*_LUMO_	–1.508	–1.507	–1.508	–1.339	–1.347	–1.348	0.059
*E*_gap_	2.052	2.043	2.046	2.265	2.256	2.257	4.581

a DFT calculations were performed with def2-TZVP, cc-pVTZ, and aug-cc-pVTZ basis sets, while CCSD­(T) results are reported with correlation-consistent basis sets (cc-pVTZ only).

**Figure 1:**
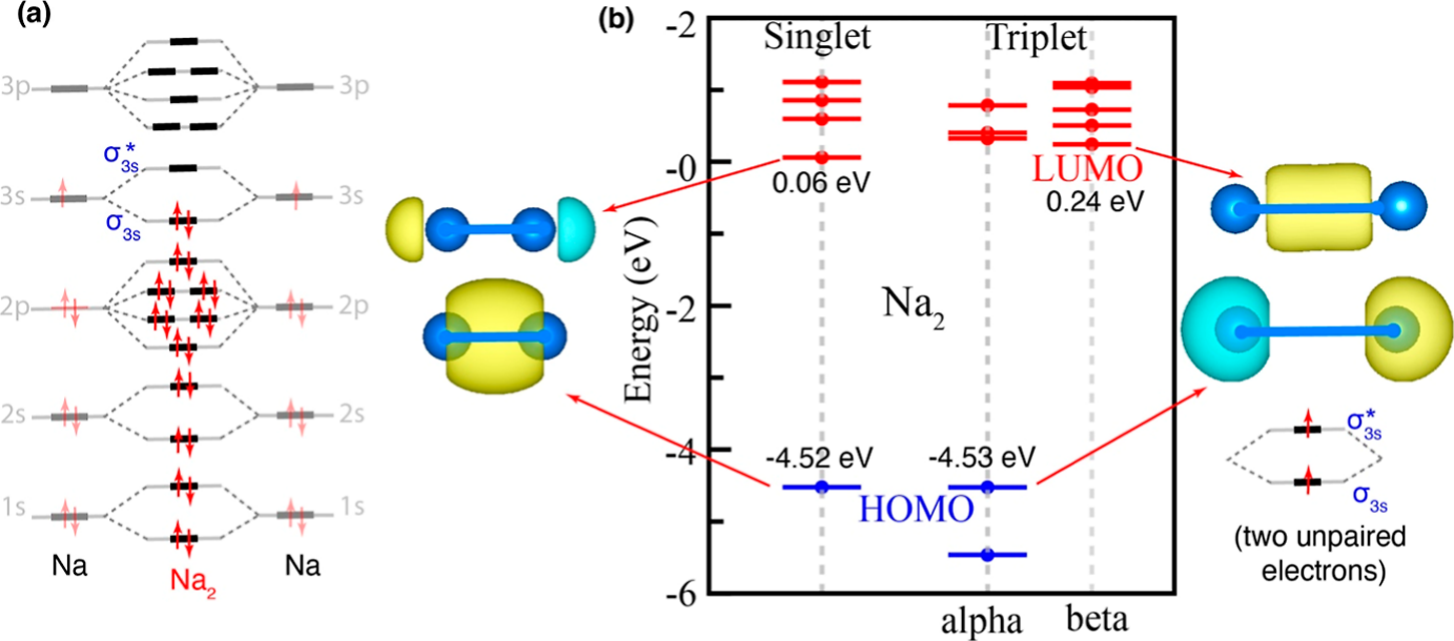
(a) Schematic representation of the molecular orbitals (MOs) of the Na_2_ dimer in the singlet state, including visualizations of the HOMO and LUMO orbital shapes, and (b) corresponding MOs in the triplet state. Atomic orbitals of the individual Na atoms are also shown for comparison.

To investigate the nature of the bonding in the Na_2_ dimer, crystal orbital Hamilton population (COHP) analysis was performed using the LOBSTER^13^ program in conjunction with DFT-D3 calculations in the Vienna ab initio simulation package (VASP).^14–16^ The integrated COHP value, reflecting bonding interactions, for the Na–Na bond in the ground state of Na_2_ was found to be ICOHP = −0.29, indicating a relatively weak covalent interaction between the sodium atoms. Moreover, we performed a reduced density gradient (RDG) analysis to characterize the nature of noncovalent interactions. The RDG versus electron density ρ plots enable the identification of different interaction regimes, including strong attractive interactions, van der Waals dispersion forces, and strong steric repulsions. The calculations were carried out using the Multiwfn software^17^ package. As shown in Figure S3 of the Supporting Information, the RDG-ρ plot for the Na_2_ dimer in the triplet state exhibits peaks around sign­(λ_2_)­ρ ≈ 0, which is indicative of purely van der Waals dispersion interactions.

Next, we examine the structure and stability of Na_2_ in its high-spin (triplet) configuration in which two unpaired electrons with parallel spins occupy separate molecular orbitals (see Figure 1). The equilibrium interatomic distances with the corresponding binding energies and the vibrational frequencies in the Na_2_ dimer in the triplet state with DFT-D3 and CCSD­(T) methods are summarized in Table 2. In the absence of long-range correction, triplet Na_2_ is not bound at the DFT level, irrespective of the exchange–correlation functional. However, once the long-range interaction, D3, is included, the binding energy (interatomic bond distance) of the triplet state against dissociation to individual atoms are −0.192 eV (4.231 Å) and −0.118 eV (4.364 Å) for the B3LYP-D3 and PBE0-D3 functionals with the cc-pvtz basis set, respectively.

**Table 2:** Calculated Bond Distances (Å), Binding Energies (eV), and Frequencies (cm^–1^) of the Na_2_ Dimer (Triplet) Using DFT (B3LYP-D3 and PBE0-D3) and CCSD­(T) Methods^a^

	DFT(B3LYP)-D3			DFT(PBE0)-D3			CCSD(T)
triplet	def2tzvp	cc-pvtz	aug-cc-pvtz	def2tzvp	cc-pvtz	aug-cc-pvtz	cc-pvtz
*R*_Na–Na_ (Å)	4.232	4.231	4.230	4.368	4.364	4.364	5.108
*E*_B_ (eV)	–0.192	–0.192	–0.193	–0.119	–0.118	–0.119	–0.030
freq (cm^–1^)	114.7	114.7	114.9	71.1	71.2	71.2	22.4

a DFT calculations were performed with def2-TZVP, cc-pVTZ, and aug-cc-pVTZ basis sets, while CCSD­(T) results are reported with correlation-consistent basis sets (cc-pVTZ only).

Since DFT is known to overbind, we have repeated the calculations using the CCSD­(T) level of theory.^18–20^ The energy, Δ*E*(*R*) = *E*[Na_2_(*R*)] – 2*E*(Na), as a function of the interatomic distance *R*, for Na_2_ in the triplet state is compared with that of the singlet in Figure 2. Note that the triplet state is higher in energy than the singlet state at all distances, although the difference narrows significantly as the interatomic distance gets larger. In the inset, we magnify Δ*E*(*R*) between 4.5 and 6.0 Å. A minimum in energy at 5.108 Å of the triplet state is clearly visible. The vibrational frequency calculated at this distance for the Na_2_ triplet is 22 cm^–1^ compared to 170 cm^–1^ for the singlet Na_2_. These values indicate that the triplet state belongs to a local minimum in the energy surface, although the binding is considerably weaker. The binding energy and interatomic distance in the spin-triplet state of Na_2_ at the CCSD­(T) level are −0.030 eV and 5.108 Å, respectively.

**Figure 2:**
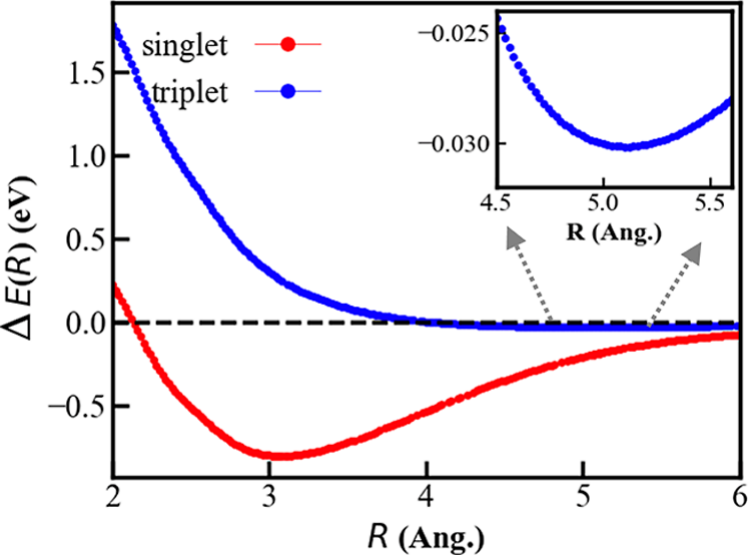
Energy, Δ*E*(*R*) = *E*[Na_2_(*R*)] – 2*E*(Na), as a function of the distance, *R*, between two sodium atoms in the spin-singlet (red) and spin-triplet state (blue), calculated using the CCSD­(T) method with the cc-pVTZ basis set. The inset shows a zoomed view around the equilibrium distance (5.108 Å).

To examine the stability of the triplet Na_2_ at finite temperature, we carried out ab initio molecular dynamics (AIMD) simulations at 300 K for 5 ps with a time step of 1 fs by using the DFT-PBE0-D3 level of theory and the VASP code. Since the energy is highly sensitive to bond breaking and formation, we tracked the total energy as a function of simulation time (Figure 3). Using the *NVT* ensemble, we monitored the energy and Na–Na bond length throughout the simulation time. Both quantities fluctuated around their steady-state value due to thermal vibrations, indicating the stability of Na_2_ in the singlet state at 300 K. The same AIMD simulation setup was applied to Na_2_ in the triplet state. In this case, both energy and bond length fluctuations were larger compared to the singlet state; however, the triplet Na_2_ remained stable up to 300 K. Recall that the binding energy of the triplet Na_2_ at the DFT-PBE0-D3 level of theory, namely, −0.118 eV, is significantly larger than the −0.030 eV calculated at the CCSD­(T) level of theory. Thus, it is reasonable to assume that the triplet state may be unstable at room temperature.

**Figure 3:**
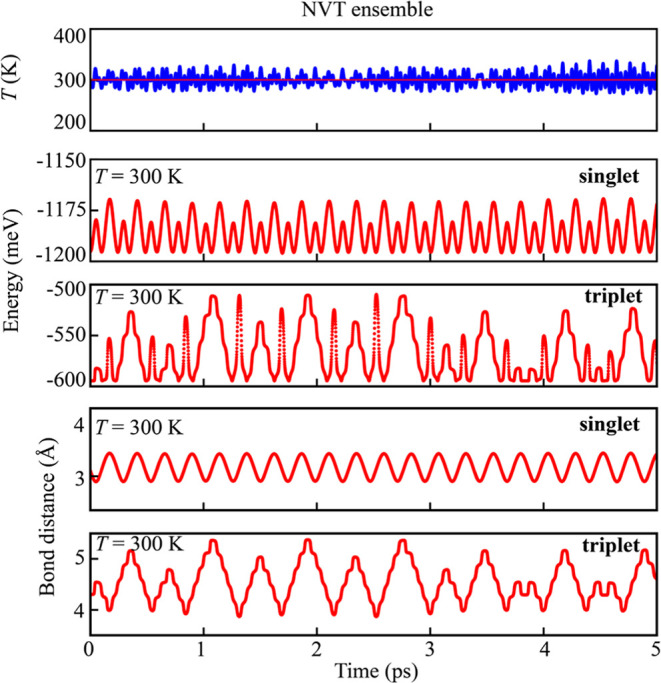
Temperature (K), energy (eV), and bond distance (Å) as a function of molecular dynamics simulation time (ps) at 300 K for Na_2_ in the singlet and triplet states under *NVT* ensemble.

To see if Li_2_ dimer can also exist in a high-spin state, we repeated the above calculations at the CCSD­(T) level with cc-pvtz basis sets. Similar to that of Na_2_, Li_2_ was also found to form in the triplet state with a binding energy of −0.046 eV and a bond length of 4.111 Å. In comparison, the ground state of Li_2_ is a spin-singlet with a binding energy of −0.567 eV and a bond length of 2.667 Å. The details of these calculations are given in the Supporting Information (Table S2 and Figure S2 of the Supporting Information).

## 4 Conclusion

We show that the high-spin state of Na_2_ lies at a minimum in the potential energy surface, which is confirmed by positive frequencies. In agreement with the experiment, the high-spin state is metastable with a binding energy of −0.030 eV and interatomic distances of 5.108 Å at the CCSD­(T) level. The binding is governed by van der Waals interaction. The advantage of the experimental technique used by Kresin and collaborators is that the cluster temperature is precisely known, eliminating any ambiguity in the measured magnetic moment. Thus, measuring the deflection of clusters formed on helium nanodroplets in a Stern–Gerlach field is an ideal technique to study the magnetism of clusters. It is hoped that this technique will be used not only to validate the prediction of metastable Li_4_ is the high spin state^2,3^ but also to study larger clusters of both nonmagnetic and magnetic atoms.

## Supplementary Material



## Notes

Total energies of the Na_2_ dimer in the triplet state using CCSD­(T) with the basis set of cc-PVXZ and aug-cc-PVXZ where (X: D, T, and Q); triplet Na_2_ total energy as a function of basis set size (X = D, T, Q, 5) using aug-cc-pVXZ, with extrapolation to the CBS limit indicated; binding energy, bond length, and vibrational frequency of the Li_2_ dimer; energy Δ*E*(*R*) = *E*[Li_2_(*R*)] – 2*E*(Li) vs distance *R* for singlet and triplet states at CCSD­(T)/cc-pVTZ; and RDG as a function of electron density ρ plot (PDF)
