# Supplementary material for: Understanding the Existence of a Na2 Dimer in a High-Spin State
Source: J Phys Chem A. 2025 Sep 2;129(40):9158–62. doi: 10.1021/acs.jpca.5c03939 (PMC12516716; doi:10.1021/acs.jpca.5c03939)
Supplement: Supplementary file 1 [file jp5c03939_si_001.pdf]

Supporting Information:

Understanding the Existence of a Na_2 Dimer in a High-spin State

Mehmet Emin Kilic and Puru Jena*

Department of Physics, Virginia Commonwealth University, Richmond, VA 23284-2000, USA

*Email: pjena@vcu.edu

Table S1 Total energies (in Hartree) of the Na_2 dimer in triplet state using CCSD(T) with the basis set of cc-PVXZ and aug-cc-PVXZ where (X: D, T, Q, 5).

X	cc-PVXZ	aug-cc-PVXZ	daug-cc-PVXZ	taug-cc-PVXZ
**D**	-323.70683	-323.70698	-323.70700	-323.70701
**T**	-323.71695	-323.71700	-323.71701	-323.71701
**Q**	-323.71836	-323.71837	-323.71837	-
**5**	-323.71829	-323.71830	-	-

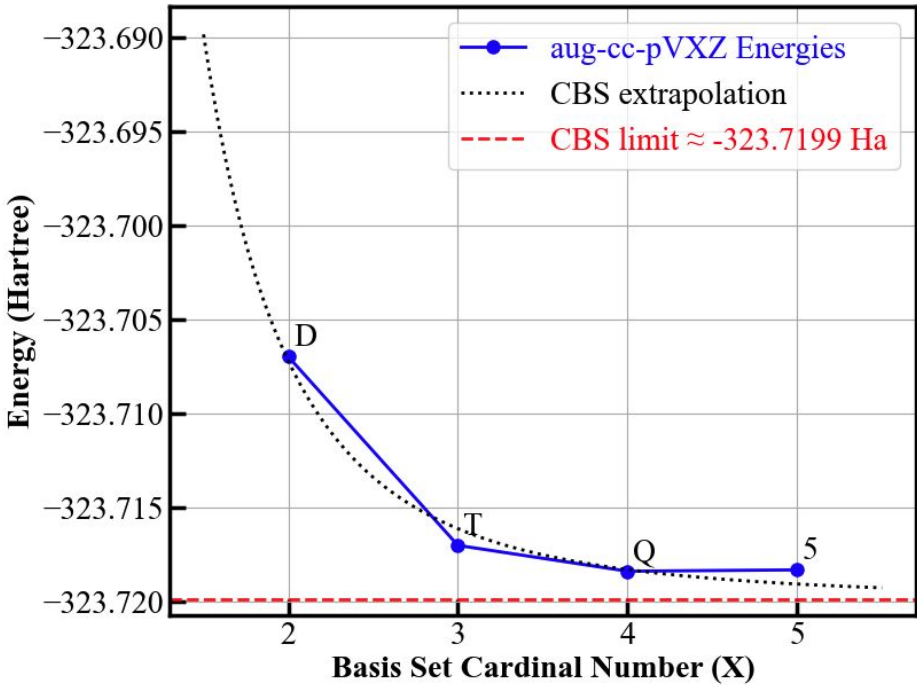

Fig. S1 Total energy of the Na_2 dimer in the triplet state as a function of the basis set cardinal number X using the aug-cc-pVXZ series (X = D, T, Q, 5). The extrapolated curve toward the Complete Basis Set (CBS) limit is shown as a dashed black line, and the estimated CBS limit is indicated by a dashed red line.

Table S2 Binding energy (eV), equilibrium bond distance (Å) and vibrational frequency (cm^{-1}) of the Li_2 dimer.

		**DFT(B3LYP)-D3**			**DFT(PBE0)-D3**			**CCSD(T)**
		def2-TZVP	cc-PVTZ	aug-cc-PVTZ	def2-TZVP	cc-PVTZ	aug-cc-PVTZ	cc-PVTZ
E_B (eV)	singlet	-0.913	-0.939	-0.942	-0.836	-0.852	-0.856	-1.058
	triplet	-0.287	-0.326	-0.328	-0.178	-0.209	-0.212	-0.046
d(Li-Li) (Å)	singlet	2.722	2.703	2.704	2.745	2.730	2.729	2.667
	triplet	3.612	3.571	3.571	3.717	3.636	3.635	4.111
Freq. (cm^{-1})	singlet	322.7	330.9	331.7	328.4	333.3	334.2	344.9
	triplet	286.3	320.1	320.6	173.7	201.3	202.3	69.3

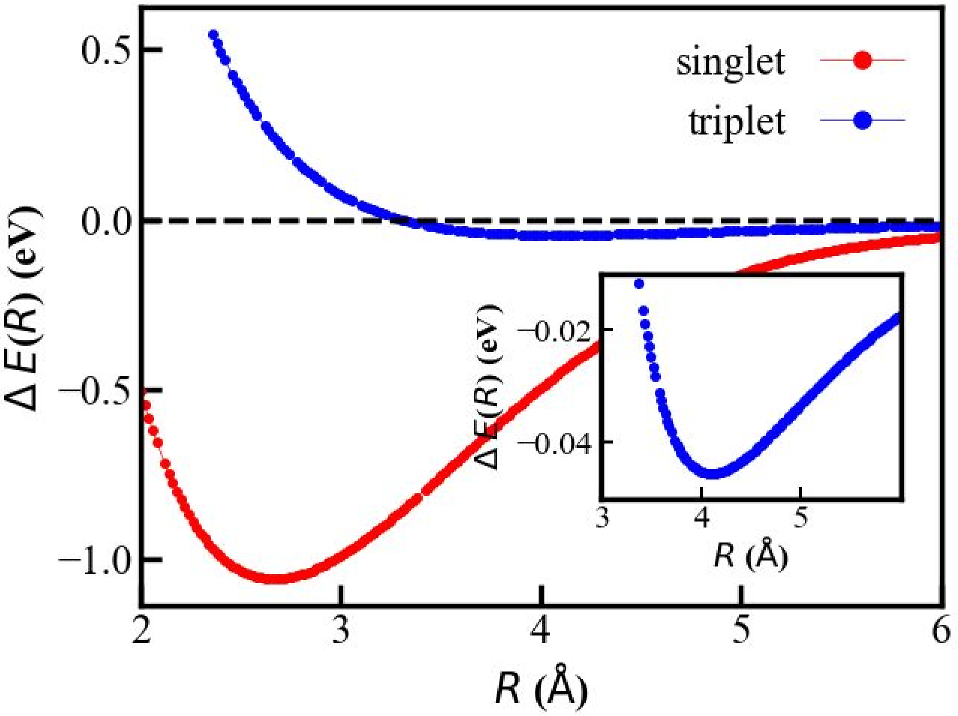

Fig. S2 Energy, $\Delta E(R) = E[Li_2(R)] - 2E(Li)$, as a function of the distance, *R*, between two lithium atoms in the (a) spin-singlet (red) and (b) spin-triplet state (blue), calculated using the CCSD(T) method with the cc-pVTZ basis set. The inset shows a zoomed view around the equilibrium distance (4.111 Å).

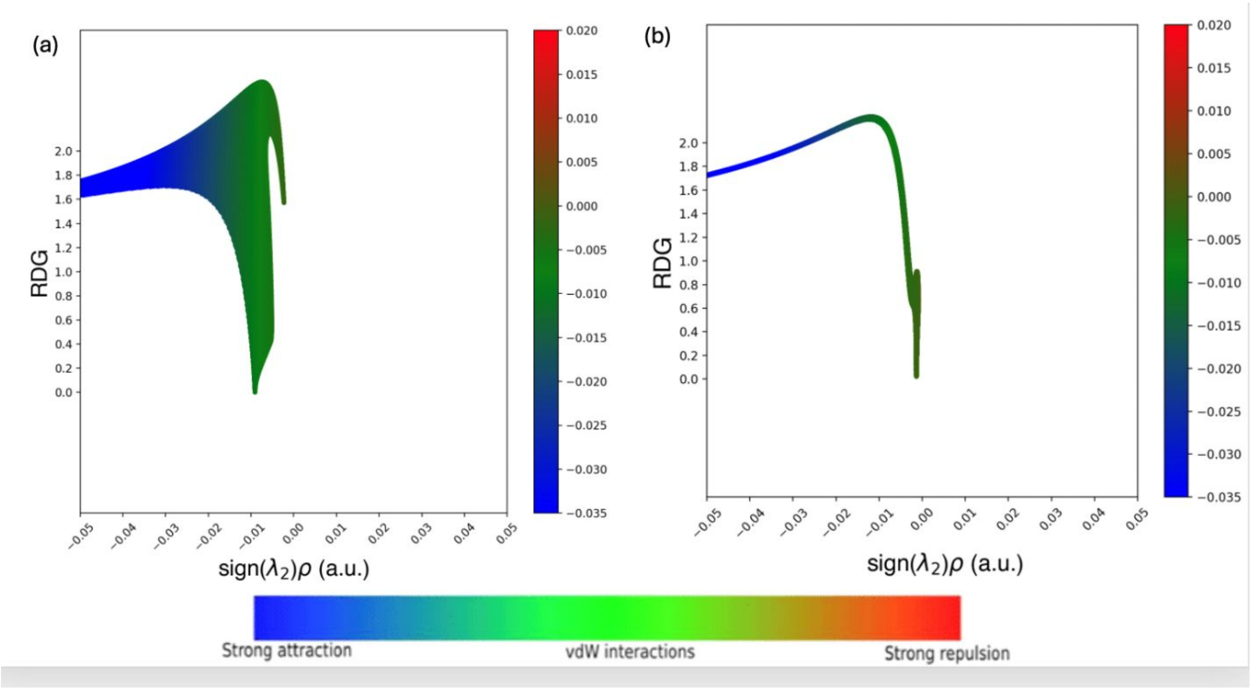

Fig. S3 RDG plot showing covalent and non-covalent bond interactions for Na_2 dimer in the singlet (left) and triplet state (right).
